# Surface Characterization of Retrieved Metal-on-Metal Total Hip Implants from Patients with Adverse Reaction to Metal Debris

**DOI:** 10.3390/ma7031866

**Published:** 2014-03-04

**Authors:** Maria Burbano, Robert Russell, Michael Huo, Robert Welch, Diana Roy, Danieli C. Rodrigues

**Affiliations:** 1 Department of Bioengineering, University of Texas at Dallas, 800 W. Campbell Rd., Richardson, TX 75080, USA; E-Mails: mxb121131@utdallas.edu (M.B.); dxr111730@utdallas.edu (D.R.); 2 Department of Orthopedic Surgery, University of Texas Southwestern Medical Center, 1801 Inwood Rd., Dallas, TX 75235, USA; E-Mails: rdrussell25@gmail.com (R.R.); michael.huo@utsouthwestern.edu (M.H.); bob.welch@utsouthwestern.edu (R.W.)

**Keywords:** metal-on-metal, adverse local tissue reaction, retrieval, pseudotumor

## Abstract

The use of metal-on-metal (MoM) total hip implants has decreased recently due to reports of high failure rates and adverse local tissue reaction (ALTR). It has been hypothesized that wear metal debris released from CoCr bearing surfaces may provoke delayed hypersensitivity reactions. The goal of this study is to evaluate the microscopic bearing surface characteristics of implants revised due to evidence of ALTR. The bearing surface of each head and cup was analyzed using multiple microscopy techniques for characterization of the surface features. The presence of severe mechanical scratching was a common characteristic found in all of the implants evaluated. Mechanical factors seemed to be the prevalent failure mode related to the appearance of ALTR with this particular set of retrieved implants.

## 1. Introduction

Artificial implants used in total hip arthroplasty (THA) are subject to friction and wear, which result in the formation of particulate debris [1]. Debris from the traditional metal-on-polyethylene (MoP) bearing components used in these procedures has been linked to osteolysis and destruction of the surrounding bone [2,3]. Therefore, alternative designs, such as the metal-on-metal (MoM) bearing components, have been introduced in the market in an attempt to reduce the formation of wear induced debris. However, these MoM bearings are subject to unique wear mechanisms and corrosion processes that can accelerate metal particle formation and release *in vivo* [4,5]. It has been reported that MoM implant design may be especially prone to the formation of larger amounts of small sized wear and corrosion products [2].

MoM systems were initially attractive because they were thought to induce the formation of a lubricating film on the surface of the implant. This protective film could lead to a reduction in wear rates in comparison to MoP bearing surfaces. Another benefit of this design was the possibility of using larger diameter heads, which decrease the risk of post-operative hip dislocation [6]. These advantages prompted surgeons to use MoM systems for total hip arthroplasties and hip resurfacing. However, MoM components have been associated with an alarming number of failures and reported patient complications. MoM bearing surfaces have been observed to release large concentrations of metal particles *in vivo*, raising the metal ion serum levels [3,6–13]. The increase of metal ions in the implant surroundings can cause inflammatory reactions in the peri-implant tissues [11].

Pseudotumors, aseptic lymphocytic vasculatis-associated lesions (ALVAL), or adverse local tissue reactions (ALTR) in the area surrounding MoM hip implants are becoming a larger subject of concern for prosthetic design companies, surgeons, and patients [9,14]. This is especially alarming because a gradual increase in the incidences of these adverse tissue reactions has been reported throughout the years [15]. In addition, the incidence of pseudotumors from THA and hip resurfacings related to MoM designs is understated, due to the fact that pseudotumors tend to be only found after revision surgery. In some patients, the symptoms of soft tissue reaction are so minimal that they do not get revised [15–17]. Recently, such has been the impact of the reactions caused by MoM implants that all the different terms used to describe the MoM lesions are starting to be encompassed into one single term: “adverse reaction to metal debris” (ARMD), which describes the formation of any mass in the body associated with the presence of a metal implant [18]. Even when correctly positioned, failure rates for MoM implant designs have been reported to reach almost 10% after seven consecutive years of their implementation [6]. Nawabi *et al*. [6] (2013) concluded that a substantial amount of these failures was due to metal debris and metallosis inside the implant environment. In a group study of THA revisions in 2008, 6% of implant recipients needed revision surgery because of metal sensitivity to the prosthesis [13].

Which variables are significant at inducing such effects are still unknown. Therefore, investigation of the possible factors affecting the performance of MoM hip implants is crucial to help mitigate problems currently observed with this particular design. Retrieval analysis of MoM systems can contribute to a better understanding of mechanisms affecting the *in vivo* performance of these implants. In this study, the surface of four MoM implants, revised due to ALTR, were evaluated using different microscopy techniques. The goal was to analyze the failure mechanisms associated with the implants and the factors that could have potentially triggered ALTR. The main area of study was the head-cup articular component of each of the implants, but modular taper junctions were also evaluated. It is hypothesized that early ARMD may be triggered by mechanical factors such as implant mal-positioning and direct MoM interaction.

### 2. Results

**Figure 1.**
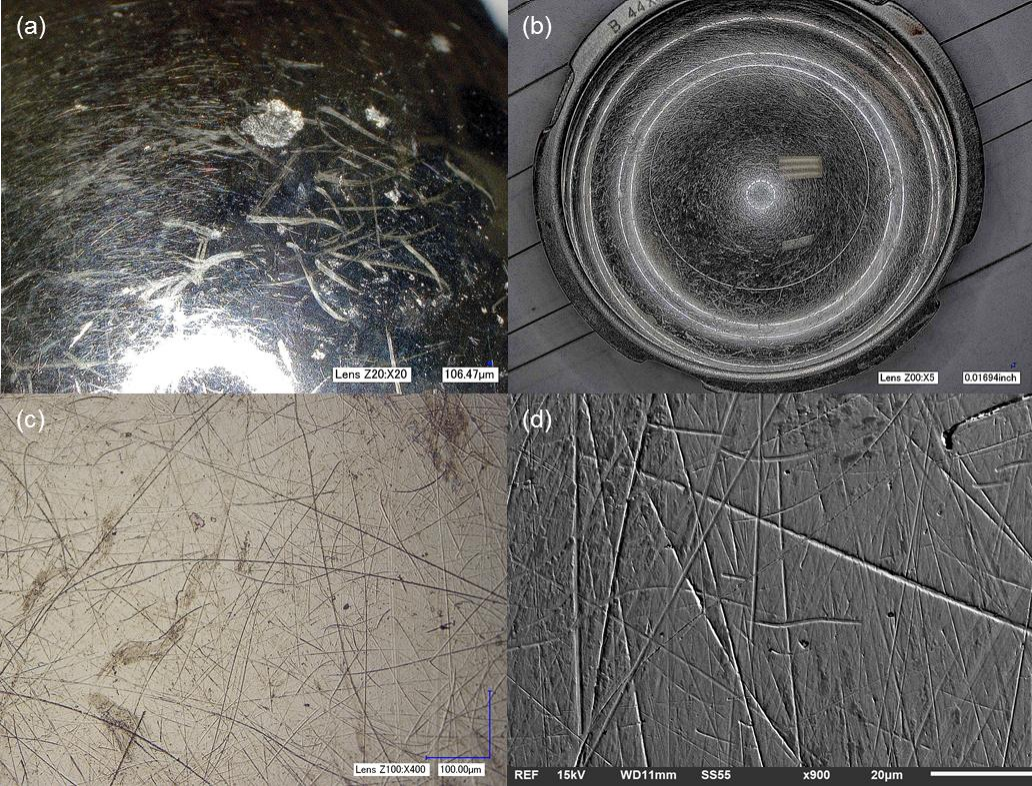
Severe scratching observed in specimen 1: (**a**) femoral head and (**b**) acetabular cup of Implant 1. Deep scratches were revealed during the analysis, which led to the hypothesis of a large amount of particle generation *in vivo*; (**c**) optical micrograph demonstrating scratch depth; (**d**) SEM micrograph showing no features other than scratching. The surface was clean of biological or corrosion products.

The four implants selected for the study presented ALTR at the time of surgical removal. All the implants consisted of CoCr MoM head-cup systems (Implants 2, 3 and 4) with the exception of Implant 1, which had a Ti6Al4V/CoCr head-cup combination. At the time of revision surgery, the head-neck taper junction was visually examined by the surgeon for any evidence of corrosion. Black debris in or near the taper junction was considered to be potential corrosion products, and the implant was recorded as having *in vivo* corrosion. Implants with no evidence of debris deposition near the head-neck taper junction, or if debris was deemed to be biologic in nature, were recorded as having no *in vivo* corrosion. Implant 1 was observed to have evidence of *in vivo* corrosion, at the time of revision surgery. However, upon microscopic inspection, the sample showed no evidence of corrosion features (pitting attack, etching, surface/bulk attack) on its surface. The articulation interface of the head and the cup was highly scratched as shown in Figure 1a,b.

The level of scratching on the head was quantified using the depth up microscope technique (Figure 1c). The total area scratched was 671 mm^2^ out of a total head surface area of 3926 mm^2^. This results in approximately 17% of the surface of the head affected by scratching. Using the clinical information, an estimated area of material lost from the scratches per year of implantation was approximately 152 mm^2^/year. Surface analysis, with the 3 different microscopy techniques, of the interfaces of this implant did not reveal any mechanisms other than scratching, as illustrated in Figure 1d. EDS analysis of areas that exhibited scratches showed similar composition to the areas of the implants with no characteristic damage ranging from 5% to 7% Carbon (C), 28% Chromium (Cr), 62% Cobalt (Co) and 3%–7% Molybdenum (Mo). Scratched and unscratched surfaces did not present a significant change in their elemental composition in all the specimens evaluated.

**Figure 2.**
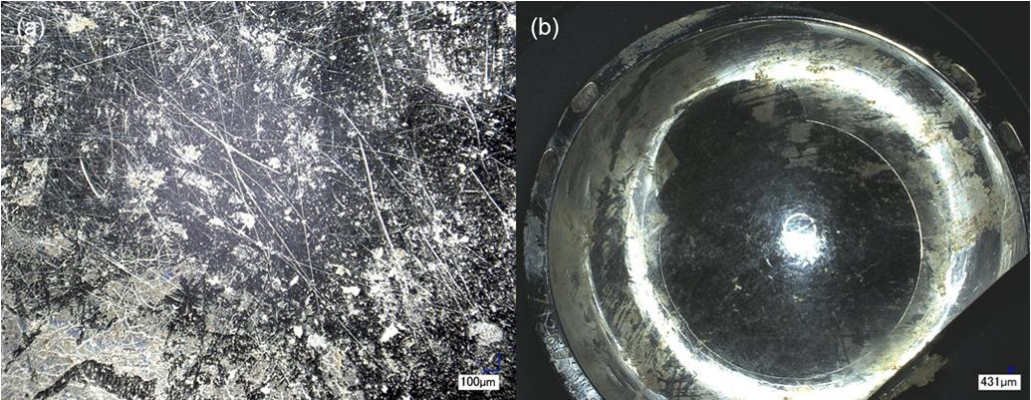

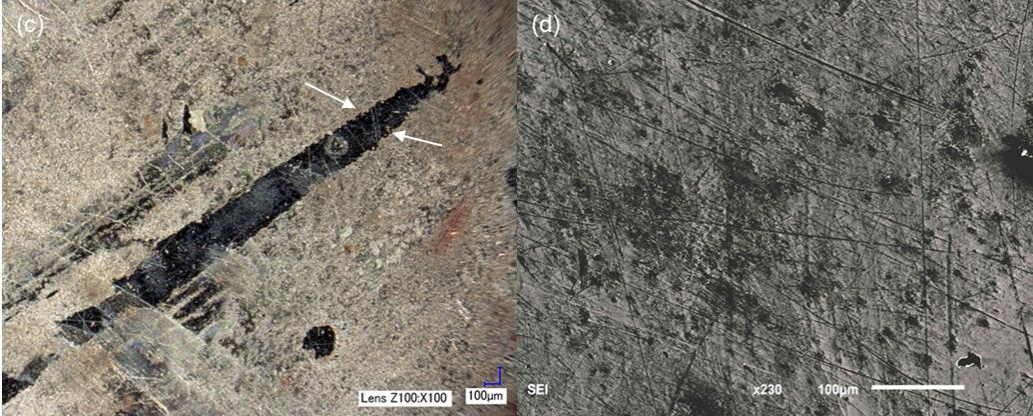
Surface condition of Implant 2: (a) optical micrograph of the scratched and debris covered surface of the head; (b) optical micrograph of the scratched surface of the cup; (c) optical micrograph showing areas of the head with high degree of biological deposition on the surface and delamination of top layers (arrows). A large amount of metal debris could be expected from this area of the implant; (d) SEM micrograph showing surface irregularities, scratches, and debris covering the surface of the head of Implant 2.

Implant 2 was recorded as having *in vivo* corrosion upon revision. Similarly to Implant 1, microscopic inspection, showed no evidence of corrosion features. However, this sample did present a large amount of debris on its surface, which appeared to be of biological origin and from wear of the top surface (delamination), as illustrated in Figure 2c,d. The level of debris was such that the head-cup ensemble was fixed in a non-rotational position. The nature of the debris was studied under SEM and EDS. A control section of the implant head with no deposition on its surface showed presence of 60% Co, 28% Cr and 5% C with traces of Mo and other trace elements. The analysis of a debris covered section of the implant showed 47% C presence, 8% Nitrogen (N) and 8% Oxygen (O); with only 23% Co and 12% Cr. In addition to these features, a large amount of scratching was observed in both the head and the cup. The results of the microscope analysis found a scratched area of the head of approximately 1309 mm^2^ in a total surface area of 7971 mm^2^. This resulted in an approximate total area loss of 350 mm^2^/year from the head-cup interface. The percentage of surface scratched on the head was 17.5%. No etching or discoloration of the interfaces of the implant was noted.

**Figure 3.**
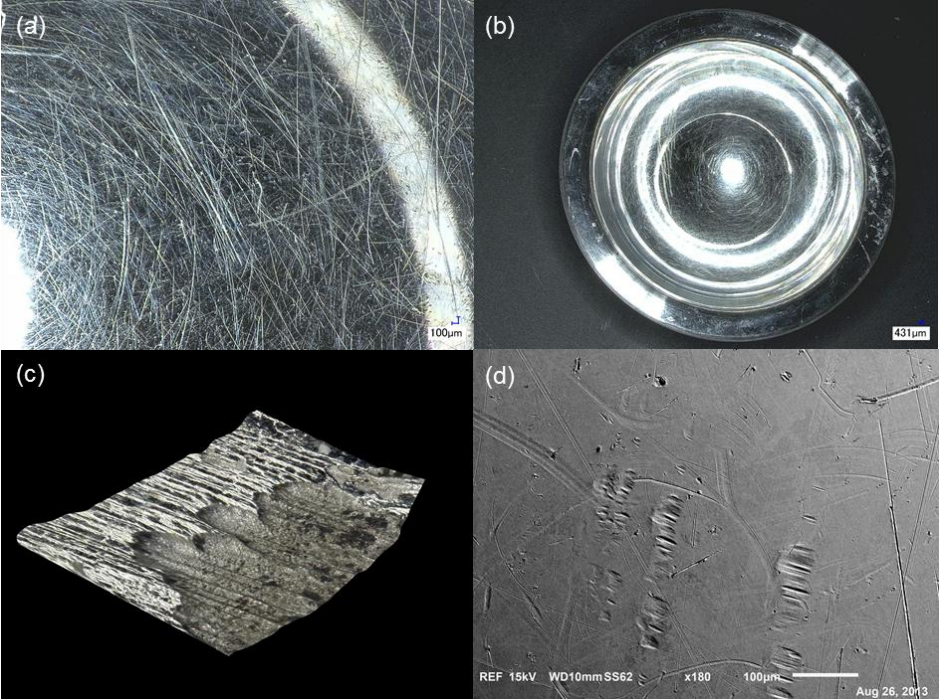
Surface condition of Implant 3: (a) optical micrograph showing the scratched surface of the head of the implant; (b) optical micrograph showing the highly scratched interfaces of the cup; (c) 3D optical micrograph revealing delamination, bulk exposure and corrosion products on the surface of the modular connection of the head female taper; (d) SEM micrograph showing scratching and fretting marks on the surface of the head.

The study of Implant 3 under digital microscopy revealed severe scratching in the head and cup areas as demonstrated in Figure 3. The total scratched area was 322 mm^2^ out of a total head area of 2532 mm^2^, which constitutes 12.7% of area scratched and an estimated area lost per year of approximately 29 mm^2^. This implant showed signs of *in vivo* corrosion at the time of surgery. Initial inspection revealed the possibility of corrosion in the implant’s head-neck modular connection due to significant surface discoloration, which was evident in the implant neck (violet and yellow discoloration). SEM analysis of the female taper of the head showed delamination of the metal surface with exposure of the metal bulk as illustrated in 3D in Figure 3c. EDS results showed the presence of 30% Co, 20% Cr, 9% Mo, 21% C, 19% O and other elements, on the non-delaminated surface of the head-neck connection surrounding the corroded delaminated area. Some small traces of Titanium (Ti) (<1%) and Aluminum (Al) (~1%) were found in this area, which were originated from wear of the implant neck (Ti6Al4V neck fretting against the female taper of the head counterpart). Analysis of the surface exposed due to delamination showed the presence of 58% Co, 27% Cr, 6% Mo, 7% C and 1% O, which confirmed bulk exposure (Co and Cr rich) due to removal of the top oxide-film containing surface. Similar to the other two implants, there was no evidence of etching, discoloration or corrosion debris present in the head and cup couple analyzed. The corrosion was only found in the female taper; the rest of the implant (surface of head and inside of the cup) only showed signs of deep scratching similarly to the other implants discussed, as observed in Figure 3a.

**Figure 4.**
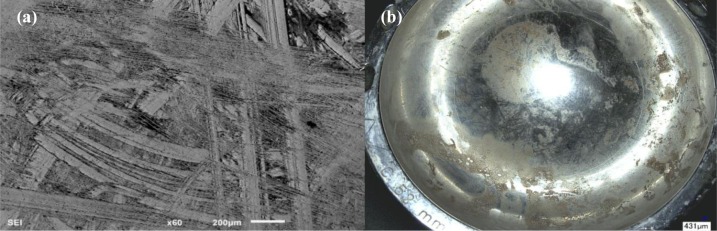

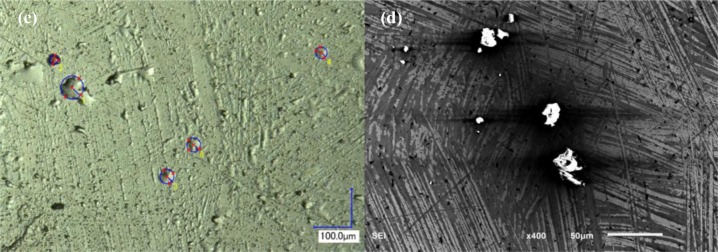
Surface condition of Implant 4: (a) SEM micrograph of the homogeneous scratching on the surface of the head; (b) optical micrograph emphasizing the debris accumulated on the surface of the cup, which was of the same composition as the debris accumulated on the surface of the head; (c) optical micrograph demonstrating the radius measurement tool in the digital microscope on irregularities found on the surface of the head; (d) SEM micrograph of another area of the head showing particles of biological nature.

As in the previous cases, Implant 4 demonstrated evidence of corrosion at the moment of revision surgery. The microscopy study of this sample revealed deep scratching similar to that observed in Implants 1, 2 and 3, as illustrated in Figure 4. The level of scratching in this implant could not be quantified due to the fine and homogeneous distribution of the scratches (Figure 4a) throughout the implant making these indistinguishable from the non-scratched surface. Biological deposition similar to that observed in Implant 2 was found on the surface of both the head and acetabular cup. EDS analysis yielded similar results as observed in Implant 2. Other than severe scratching of the implant head and cup couple, there were no signs of surface cracking, corrosion, or etching in the analyzed areas of this specimen.

## 3. Materials and Methods

### 3.1. Implant Selection

A set of 25 implants was obtained, under University of Texas Southwestern Medical Center (UTSW) IRB approved protocol, from revision surgeries of patients who underwent THA. A single fellowship trained adult reconstructive surgeon performed all the surgeries. In order to protect patient information, the implants were identified according to their date of explantation. The implants were of different designs, sizes and brands; and they were retrieved due to a range of clinical reasons including acetabular loosening, loss of stem fixation, pain, adverse tissue reaction, *etc*. The duration of implantation was not homogenous either, ranging from 2 months to 296 months of service *in vivo*. Out of the 25 samples received and evaluated, 4 MoM implants were selected based upon their metal-on-metal design, metallic composition, and the presence of ALTR as reason for revision. A summary of clinical data per implant is shown in Table 1.

### 3.2. Specimen Preparation

All the implant specimens were subjected to autoclave sterilization post-surgery. Upon receiving, the specimens underwent a general evaluation for assessment of implant conditions and gross features present on the surface. Initial observations were recorded per implant component. After this initial inspection, all the implants were cleaned using soap water and ethanol for removal of remaining debris. For the cleaning, all implants were first submerged in anhydrous ethanol (Fisher scientific, Waltham, MA, USA) for 48 h. Following submersion, the implants were rinsed with deionized water and set to dry. Two of the implants were subjected to additional cleaning because of the presence of accumulated biological matter adhered on their surfaces. This additional cleaning step was performed in an ultrasonic bath (Bransonic Series CPX3800H, Danbury, CT, USA) with the samples immersed for 1 h in neutral soap solution. The specimens were then dried by hand and were finally subjected to a final ultrasonic cleaning step in ethanol. The cleaning was effective in removing biological materials and loose particulate deposited on the surface, better revealing surface features resulting from any potential corrosive or mechanical processes.

### 3.3. Surface Analysis

Following cleaning, the specimens head and cup were subjected to microscopy for determination of particular areas of interest exhibiting signs of degradation. The head/neck modular taper region was also inspected in all the specimens. Surface analysis was performed under low (0×–5×) and high (100×–1000×) magnifications using a Keyence Digital Microscope VHX-2000 (Osaka, Japan). The microscope software features enable identification of surface characteristics such as scratching, pitting, and corrosion. Areas with apparent evidence of corrosion or biological debris were marked and sent for sectioning.

The marked and cut sections of the implants (CoCr heads) underwent surface analysis with Scanning Electron Microscopy, SEM (JEOL, JSM-6010 LA, Peabody, MA, USA). The specimens were analyzed with multiple magnifications and with accelerating voltages from 10 to 20 kV. The SEM was equipped with an Energy Dispersive X-ray Spectrometer (EDS), which provided the mass and elemental composition of the materials bulk and oxide layer. The primary purpose of the EDS analysis was to reveal the composition of the marked areas to determine whether the features observed consisted of biological products or were of metallic nature. The observations were correlated with clinical data. For the EDS analysis, areas of the implant with no signs of surface damage (such as scratches, pitting, cracks, biological products, delamination, discoloration) were surveyed and considered as baseline for comparison with areas that exhibited characteristic features. All the samples were composed of the following elements: Cr, Co, Mo, C and O. The mass percentages of these elements, taken from areas of the implant with no damage, were recorded as the control measurements in order to identify the presence of corrosion or biological deposits. Approximately 5–10 measurements were taken from each area of interest. Elemental percentages for each sample were calculated from the average of the measurements taken.

**Figure 5.**
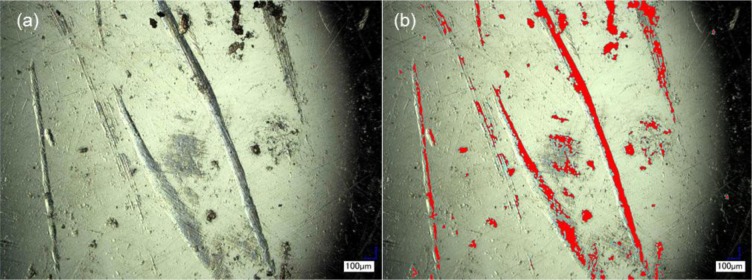
Image of the curved surface of the head of one of the selected CoCr implants taken using the depth-up feature: (**a**) selected area for analysis; (**b**) example of technique used for determination of scratch areas, highlighting some of the scratches present on the surface. The actual analysis took all measurable scratches into account that were present on the surface.

Highly scratched surfaces were detected during analysis; therefore in order to characterize and quantify the level of scratching, a 3D depth up and area measurement feature in the digital microscope software was used to make an estimate of the total scratched area (using length and depth dimensions). The 3D depth up feature allows for the capture of a full focused image across a curved plane. With this feature a set of images, with modified lighting and texture that highlighted the scratches, were obtained for each implant as illustrated in Figure 5. The scratch was defined as all slits that were deeper than 1 μm and thus detectable by the microscope. However, this feature offers a limitation on the level of curvature that can be brought to focus while still allowing the appropriate lighting for scratch area measurement. Due to this, only the head component of each implant was analyzed under this technique. The area measurement feature can select particular areas on an image based on the brightness and darkness contrast or color difference in the individual image. It provides the overall area of the image and the value of the area selected. Using this feature, the highlighted scratches were selected and the percentage of the area scratched in relation to the total image area was obtained, as shown in Figure 5b. This process was repeated with all the images. The average of the different percentages for each implant was calculated and used to make an estimate of the total area scratched in the entire head interface. This measurement was then used to roughly estimate the area of material lost per year by dividing the total scratched area over the length of implantation.

## 4. Discussion and Conclusions

The purpose of the study was to describe the bearing surface characteristics of MoM implants, selected from a large pool of specimens, showing evidence of ALTR upon revision surgery. In the overall set of implants, processes such as scratching and accumulation of biological debris were observed in more than half of the cases. Chemical attack, on the other hand, was not present in this specific set of retrievals, except for the head-neck taper connection in Implant 3 (Figure 3). Events such as surface etching, discoloration, inter-granular corrosion, or fretting corrosion signs were not observed in the head and cup components of the MoM implants evaluated, or in the head-neck modular junction of the Implants 1, 2 and 4. The lack of evidence of corrosion in the interfaces evaluated suggests that mechanical factors, such as wear and scratching, may be a major contributor in triggering ALTR. This finding is corroborated by other studies that have found that the main reasons for revision surgery are mechanical in nature, including acetabular loosening, femoral loosening, fracture and mal-positioning [19]. In this study, the principal reason of concern in all of the implants evaluated was the level of scratching found across the surface of the head and cup articulation interfaces. The remarkable level of scratching observed indicates that these implants released a large amount of metal particles *in vivo*. Studies have shown that patients with CoCr MoM implants present higher serum levels of Co and Cr [7,20]. Severe scratching and consequent wear and metal ion release into the body may have been the principal cause for the formation of peri-implant tissue reaction in the specific cases evaluated. These results appear to relate the ALTR observed to an ARMD.

An individual analysis of each of the samples is necessary to characterize the different factors that lead to the formation of an ARMD. In the case of Implant 1, it was found that the cup had an abduction angle of 57° and a head of 44 mm in diameter. These characteristics classify the head as large and the cup abduction angle as outside of the *Lewinnek Zone* [17]. It has been reported that MoM soft tissue reactions are more likely to be found in hip prostheses with heads between 38 and 49 mm of diameter and with cup abduction outside of the *Lewinnek Zone*, which is defined between 5°–25° for anteversion and 30°–50° for abduction [17,21]. In a study of 2600 cases of hip resurfacings, Gross *et al*. [22] found a low incidence rate of pseudotumors, ranging from 0.1% to 1.8%. However, all of the failures that presented pseudotumors had small head components (less than 48 mm) and abduction angles greater than 50° [22]. Acetabular inclination is thought to be an important factor that contributes to increasing wear rates and metal particles, which increases the probability of developing a pseudotumor [21,23,24]. However, some studies have shown that there is not a significant correlation between metal serum levels and angle of cup inclination [25,26]. Thus, both studies acknowledge that accurate cup positioning lowers the possible risk of implant failure. The need for revision of Implant 1 was most likely due to its high cup abduction angle, which led to an uneven distribution of forces and stresses on the implant, inducing the observed scratching. The estimated surface area loss due to scratching (17%) is significant and may indicate that a high amount of metal ions and debris was released from this interface. Such an influx of foreign metal ions into the body may have led to the formation of an ARMD.

From studying the surface of Implant 2, the nature of the debris covering the surface of the prosthesis was likely biological, a probable result of bodily fluids entering the rotational areas of the implant. The reason for revision of Implant 2 is similar to the mechanism observed with Implant 1, likely related to its highly scratched surface (Figure 2) that released metal particles into the surrounding soft tissues, causing an inflammatory reaction and the development of ARMD. The scratching in this implant was characterized and resulted in a 17.5% of area scratched.

Upon investigation of Implant 3, optical microscope, SEM and EDS analysis confirmed the presence of corrosion on the female taper of the head-neck modular connection. The modular head-neck interface underwent fretting-crevice corrosion, which likely generated titanium ions and debris that deposited into the head-cup articulation accelerating wear [8], and in this particular case, leading to the formation of ARMD. The modularity of hip implants allows for small displacements in the individual modular connections that can induce fretting-crevice corrosion. It has been reported that main concerns related to head-neck tapers are their tendency for corrosion and accelerated wear, especially with mixed metals, thus releasing large concentration of particle debris [27,28]. A similar result was found in a recent case report that showed the formation of a pseudotumor in a 72 years-old patient as a result of corrosion and wear products that arose from the modular head-neck interface of a hip hemiarthoplasty [29]. In addition to corrosion observed in the head-neck area, the high level of scratching found on the surface of both the head and cup (Figure 3) also contributed to the formation of wear particles that resulted in the ARMD. The scratching level was quantified, as in the previous implants, revealing a 12.7% area scratched.

In the case of Implant 4, SEM and EDS revealed similar results as in Implant 2, that the debris covering its surface was biological in nature, rich in C, O and N. Implant 4 had the lowest implantation time out of the other 3 samples, demonstrating that the formation of soft tissue reactions does not require extended periods of time of metal ion release into the body. The high level of scratching observed on the interfaces of this implant (Figure 4) could not be quantified due to the homogenous scratch coverage. The surface showed absence of any other features such as delamination, etching or cracks. None of the head-cup interfaces presented discoloration, fretting marks, bulk exposure, or cracks, which are features typically associated with fretting-crevice corrosion [30], confirming the absence of corrosion products on the articulation surfaces. Implant 1 had a Ti6Al4V cup in contrast to the other three implants that had a CoCr alloy cup. The Ti alloy is softer than CoCr, and therefore could be more susceptible to scratching than CoCr interfaces. However, qualitative measurements indicated that both alloys can be subjected to similar degree of scratching.

Implants are designed with tight tolerances to prevent materials to infiltrate between the contacting surfaces, such as the head and cup. Still, bodily fluids filled with ions and gas and other molecules manage to get into those enclosed areas. This infiltration may have caused the implants to be covered with debris that likely impaired their functionality. The debris contributed to initiate scratching and wear of the articulating surface of the specimens. The scratching led to the formation of metal particles, which induced a three-body wear mechanism. Both the biological fluid and the generated particles created a positive loop that was responsible for further damaging the surface of the implants. Third-body wear could have also come into play, given bone fragments can be produced during impaction of the implant, which may penetrate the head/cup interface generating scratches.

This study has limitations, such as the small sample size analyzed and the fact that some of the implants did not have complete clinical information available. However, the aim was to discuss possible failure modes associated with MoM implants and their correlation to the formation of ARMD. Restraints in the ability of both the optical microscope and the SEM to fully characterize curved surfaces and the availability of only one method for the detection of scratch-induced mass loss of the interfaces evaluated were also sources of limitations. However, this study has the goal and significance of highlighting possible contributing factors to failure mechanisms in MoM hip implants and analyzing the reasons for formation of ARMD. Further analysis should look into the possibility of not only the MoM implant design to be the main perpetrator of ARMD, but that also the modular factor of certain designs can increase the possibility of these tissue reactions.

## Figures and Tables

**Table 1.** Implant information and clinical data.

Implant ID #	Head diameter (mm)	Cup/head material	Cup Abduction	Duration (months)	Reason for revision
1	44	Ti6Al4V/CoCr	57°	53	Aseptic loosening
2	58	CoCr/CoCr	40°	48	Pain
3	31	CoCr/CoCr	37°	134	Pain
4	46	CoCr/CoCr	45°	30	Pain
